# Precision medicine at the crossroads

**DOI:** 10.1186/s40246-017-0119-1

**Published:** 2017-10-11

**Authors:** Maynard V. Olson

**Affiliations:** 1 Department of Medicine, University of Washington, Seattle, Washington 98195 USA; 2 Department of Genome Sciences, University of Washington, Seattle, Washington 98195 USA

## Abstract

There are bioethical, institutional, economic, legal, and cultural obstacles to creating the robust-precompetitive-data resource that will be required to advance the vision of “precision medicine,” the ability to use molecular data to target therapies to patients for whom they offer the most benefit at the least risk. Creation of such an “information commons” was the central recommendation of the 2011 report *Toward Precision Medicine* issued by a committee of the National Research Council of the USA (Committee on a Framework for Development of a New Taxonomy of Disease; National Research Council. Toward precision medicine: building a knowledge network for biomedical research and a new taxonomy of disease. 2011). In this commentary, I review the rationale for creating an information commons and the obstacles to doing so; then, I endorse a path forward based on the dynamic consent of research subjects interacting with researchers through trusted mediators. I assert that the advantages of the proposed system overwhelm alternative ways of handling data on the phenotypes, genotypes, and environmental exposures of individual humans; hence, I argue that its creation should be the central policy objective of early efforts to make precision medicine a reality.

## Introduction

In human genomics today, one question looms above all others. How are we going to handle data on the phenotypes, genotypes, and environmental exposures of individual humans [1]? These data are already the lifeblood of our field and will play an increasingly dominant role in human-genomic research for decades, if not centuries, to come. We already collect these data in quantities that were unthinkable a few years ago, and a tsunami of new data will soon be upon us. Indeed, this metaphor is inadequate. Tsunamis are discrete, rare events that do a lot of damage and then recede. Survivors bury the dead, pick up the debris, beef up seawalls, and get on with their lives. In contrast, we are not dealing with a one-time event: the flux of data about human phenotypes, genotypes, and environmental influences will just keep growing, exponentially or super-exponentially, for the foreseeable future. Furthermore, the basic character of these data will differ greatly from those that human genomicists have gathered in the past. We need a strategic plan for managing these data, and it is increasingly obvious we lack one.

Geneticists and genomicists like change and have a good record of adapting to it. Consider the rapidity with which recombinant-DNA and genomic techniques allowed human geneticists to solve longstanding problems in the 1980s and 1990s. In that era, much of the energy of human geneticists went into exploring local features of the human genome in cottage-industry fashion. Once the whole genome had been sequenced, the energy once expended mapping out megabase-pair-sized regions, no easy task in the 1980s, was freed up for more scientifically rewarding endeavors. An optimist might imagine a similarly smooth transition from the current era, in which human genomicists and their collaborators expend enormous energy enrolling patients in one-off research studies, to an era in which huge data sets containing genomic, phenotypic, and environmental data on millions of recontactable people become widely available. In this essay, I argue this will not happen unless we make a big push now to create a true information commons. Inaction or misdirected actions pose an existential threat to the open-science traditions of human genomics. In what follows, I elaborate on this alarmist view and sketch a path forward that offers a more promising future for all stakeholders than the path we are now on.

### Lessons of history

From its inception, genomics has had a split personality. In one guise, genomics is an interdisciplinary field that brings a distinctive point of view and set of increasingly powerful techniques to the analysis of diverse problems in basic biology. From this perspective, although some genomic data have utilitarian value, pursuit of practical applications is not the field’s *raison d’être*. In another guise, genomics is on a fast track to providing humans with long, healthy lives and making a few venturesome genomicists rich. During the dot-com bubble of the late 1990s, which was accompanied by a biotech bubble, a company developing gene-analysis platforms ran an advertisement showing a double helix rising up through brightly colored mist, accompanied by the slogan “At the top of this ladder is a world without disease” [2]. This slogan is just plain stupid. Not only do we, like all other mortal agents, lack the ability to banish disease from the human condition, we risk creating new niches for disease at a faster pace than we attenuate or eradicate old ones. There is a reason why pneumonia was once called “the old man’s friend.” Advances in medicine have always involved a delicate balance, easily gotten wrong, between increasing health and increasing disease, the latter effect arising when heavily medicalized lives are extended beyond reasonable limits. The more effective genomic medicine becomes, the more vulnerable it will be to the latter trap.

This risk notwithstanding, human genomics has managed its dual personality tolerably well. There has been some embarrassment when mostly friendly critics ask “Where are the goods?” but these queries have been little more than polite requests that some of our noisier colleagues lower their voices. We have retained public support because many people recognize that genomics has already delivered a lot of goods, both directly and indirectly. Direct benefits have been realized in areas such as genetic testing and DNA forensics, while indirect ones have accrued on a much broader front. The indirection of most of genomics’ contributions to biology is a natural consequence of what genomics is. Genomicists solve few problems on their own, but they empower all biological research at the molecular level and even much research at higher levels of biological organization. A plausible analogy can be made with computer science, most of whose contributions to scientific knowledge and societal wellbeing are also indirect.

Of course, from time to time, tension between genomics’ two personae has flared up. Controversies about the wholesale patenting of gene sequences are one example [3]. The conflict between public and private sector participation in the Human Genome Project (HGP) is another [4]. The mantra of the major private sector participant in the HGP was “Discovery can’t wait!” Evidently, what discovery could not wait for were the processes that communities of scientists rely on to accumulate broadly accessible knowledge, insure high-quality standards, and keep tabs on each other’s behavior. Scientists such as myself, who argued for balancing the desirability of moving quickly in the HGP with that of preserving the Mertonian virtues of communalism, universalism, disinterestedness, and organized skepticism, were attacked for being indifferent to the suffering of patients whose treatment might benefit, in some unspecified way, from turning the HGP into a crash program [2, 5].

Those were yesterday’s battles, which were largely won by proponents of open science. In the USA, we now have a unanimous Supreme Court decision that prevents the use of patent law to restrict free use of bulk genome sequences [6]. We have a high-quality reference sequence of the human genome that is accessible to all, large public repositories of human genetic variation data, and a robust research community that continually adds value to these resources through peer-reviewed publications in the open literature.

### The present dilemma

Threats to the open-science tradition in human genomics now come from several directions, particularly with regard to research on human phenotype-genotype correlations. Since research in this area is likely to dominate human genomics for the foreseeable future, these threats demand our closest attention. Here is a brief outline of the reasons the open-science traditions of our field are under threat:

- *Exaggerated concerns about patient privacy.* Privacy concerns are the main obstacle to an open-science approach to the study of human phenotype-genotype correlations. An elaborate web of laws, regulations, cultural practices, and entrenched beliefs walls off rich data sets about individual humans from the research community. Given the long history of genetic exceptionalism in attitudes toward privacy [7–9], there is no way to undo or redo past decisions. The only path forward is to empower patients to choose the level of privacy they are comfortable with and then attempt to persuade them, one at a time, to make choices that will allow research to go forward. I outline below how such a system might work and argue that there are reasons to hope that patients and their families will prove more willing to contribute their data to an information commons than many believe they will be. The potential for abusive efforts to identify research participants through their genetic profiles or information in their medical records will only grow in the future. It cannot be eliminated. We simply need to bring genetic privacy into the same tent that houses the escalating privacy concerns that permeate modern life, not treat them as something unique unto themselves. People will always vary greatly in the level and nature of their privacy concerns. Our current impulse to impose paternalistically one-size-fits-all ways of addressing them are both futile and, arguably, unethical.
- Increasing reliance of the biomedical research community on large health care delivery systems. The only feasible way of acquiring phenotypic data on millions of individuals is by capturing information collected in the ordinary course of clinical care. With patient consent and supplementary funding, these data could be augmented with genotypic and phenotypic assessments that are safe and easily carried out, even when not medically indicated. Large health care delivery systems are the only organizations that can house and conduct this activity. No other approach will be affordable, sustainable, or logistically feasible. It is one thing to gather supplementary molecular data on tissue samples collected in the ordinary course of clinical care but quite another to imagine that millions of people are going to participate regularly in a parallel system of phenotypic assessment for much of their lives. A three-way marriage between patients, researchers, and health care delivery systems will be awkward, but it offers the only path forward. The rule in building relationships that make sense for all parties—even if there is little mutual attraction or even natural compatibility among them—is “one step at a time.” I outline below an incremental approach that would observe this rule and be a better use of public resources than hurried efforts to enroll a lot of patients in inadequately designed, underfunded, long-term studies.
- *Increasing reliance of everyone on the information technology* (*IT*) *industry*. The days are gone when human genomicists should be building and maintaining their own information technology infrastructure. Major resources are presently wasted supporting legacy systems that made sense in the 1990s but should now be retired. At a time when large corporations are downsizing IT departments and outsourcing computing needs to companies that actually know how to deal with the twenty-first century data, most research centers cling to do-it-yourself operations. The good news is that computing, like sequencing, is getting cheap. The bad news is that the growing gap between the computing capabilities of typical research centers and those of the IT industry risks driving population-scale genomics into the arms of this industry without any strategic plan to maintain public control over the data. Health care delivery systems already have the relationships with patients that are the sine qua non of population genomics and have reasons of their own to want to increase their involvement in research on medically relevant phenotypes, genotypes, and environmental influences. IT companies are the only entities capable of managing the data. Hence, for advocates of open science, the obvious risk is that players in these two sectors will join forces, relegating both academic researchers and the public interest to the sidelines. Of course, academic researchers will still be consulted when it suits the needs of private sector genomic companies, but the consultations will occur on their terms, not ours.
- *Self-interest*. So far, I have emphasized the external forces favoring privatization of population-scale genomics. Now, I turn to internal practices within human genomics that contribute to this threat. Communities that aspire to make the world a better place should always start by looking in the mirror. Human genomics has a mixed record on data sharing, and my proposals will be no more welcome to some of my academic colleagues than to many entrepreneurs seeking commercial opportunity in the marriage of genomics and medicine. Human genomics is a fusion discipline forged from two fields with different data-sharing practices. Genomics largely grew out of model-organism biology, in which sharing of strains, protocols, and data have long been the norm. In contrast, human genetics has never had a strong data-sharing tradition: human geneticists “own” their patients and guard access to them zealously. The reasons for these divergent traditions are easy enough to understand. Researchers develop relationships of mutual trust with human research subjects but not with yeast strains and mouse lines. Furthermore, access to patients and patient data is now restricted by a tangle of bureaucratic, regulatory, and legal constraints. Nonetheless, self-interest is a more formidable obstacle to data sharing than laws like the Health Insurance Portability and Accountability Act in the USA [10]. Careers are often built by enrolling valuable patient populations in research studies and then permanently sequestering them from competitors. As the sizes of these populations increase, it is not just individual careers but whole research bureaucracies that sometimes appear more focused on controlling access to their valuable patient resources than solving scientific problems. If we are to create an information commons containing comprehensive data about individual research subjects, this system must be reformed.

These bullet points frame the present dilemma. The task of addressing any one of them would be daunting enough, but, given the way they reinforce each another, advocates of maintaining and enhancing an open-science tradition in human genomics will need to address all at once. Perhaps the impulse to do so is simply Quixotic. What motivates me is a dystopian vision of the way research in our field is likely to evolve if we let current momentum carry us where it will. For starters, a business-as-usual scenario will increasingly marginalize academic researchers in human genomics, a community already under stress. Academic researchers will find that the legal and regulatory systems that once protected their proprietary access to particular patient populations can be deployed to far greater effect by privatized entities formed through alliances between health care delivery systems and the IT industry. These organizations will hire the legal staffs and lobbyists they need to lock down everything they control that has commercial value. Federal agencies such as the National Institutes of Health (NIH) in the USA, created to represent and promote the public interest in biomedical research, are also at risk. The regulatory power of the NIH depends on the ability of its Institutes to make grant funding contingent on agreement by researchers and their institutions to do things the NIH way. The NIH’s Institutes are not true regulatory agencies: they lack the experience, staff, and standing to oversee a sprawling, privatized research enterprise that is deeply embedded in a multi-trillion-dollar-per-year industry. Agencies that do regulate that industry will acquire de facto control over most research as an indirect consequence of their responsibilities in regulating patient care. Programs such as the Precision Medicine Initiative (recently rebranded the *All of Us* Research Program), which was launched with great fanfare by an administration no longer in office [11], are likely to end up under-funded, under-powered, over-regulated, and unable to deliver on their promise. Legislators who would welcome increasing privatization of biomedical research and attendant tightening of the link between research and commercial opportunities will see the NIH’s mission as increasingly irrelevant to their priorities. The situation in the USA will differ less from that in countries with government-sponsored national health care systems than one might imagine. Federal and state governments already pay for two thirds of health care in the USA with most of the money coming from Washington, D.C. [12].

Not everyone, even within academia, will consider the future scenarios I have sketched as undesirable. Hence, before proposing an alternative future for population-scale genomics, I will briefly defend my view that science, industry, and society would all benefit if key players take bold action now to design a more open future for population-scale genomics. My argument rests on the societal value of defending a line, or perhaps zone is a better descriptor, separating precompetitive and proprietary knowledge. I think all players—science, industry, and the larger society—win if we give careful consideration to the types of data and knowledge that belong on one side of the line or the other. Science wins when researchers have unencumbered access to as much data and knowledge as possible since, at least on a time scale of decades, the messy processes associated with open science overwhelm the transient advantages that sometimes accrue to closed organizations. As private sector partisans like to point out, academia cannot match the ability of closed organizations to raise capital, build infrastructure, manage skilled workforces, and act decisively. However, one cannot manage one’s way to identifying and exploring the “unknown unknowns” that stand in the way of our ability to achieve better health. Industry wins because commercial organizations can focus on what they do best: determining whether or not seemingly good ideas are market-ready and, if so, shepherding them through the research and development pipeline that actually delivers drugs, diagnostic tests, and medical devices from bench to bedside. Society wins for the simple reason that practical advances in the modern world are tightly linked to expanding knowledge, and open science has a large edge over other systems as a way of learning new things. For these reasons, I am confident that bold action to create a precompetitive information commons would be a win-win-win proposition for science, industry, and society.

## What should be done

Questions about what should be done are ultimately about power: in whose hands should it reside and how should it be deployed? I will borrow my answer to this question from the title of a paper by Sharon and Patrick Terry [13], two tireless advocates of increased data sharing in biomedical research: “Power to the People!” Empowering the people on whose cooperation population-scale genomics will depend, and for whose benefit it should be carried out, would solve a whole nexus of problems. I have been referring to these volunteers as “patients” and “research subjects,” but, first and foremost, they are just us. The volunteers who will have to contribute DNA, images of their internal organs, samples of their body fluids, and access to their electronic medical records to the commons are just us, all of us.

We need to promote a new social contract between patients and the health care systems on which they depend. Susan Desmond-Hellmann, who co-chaired the committee of the United States National Research Council that issued the report *Toward Precision Medicine*, a committee on which I served, articulated the need for this new contract in an editorial in *Science Translational Medicine* [14]:

I believe that the most important requirement for the new knowledge network envisaged by the *Precision Medicine* report is that it be driven by patients. Indeed, it is patients who particularly understand the potential value of a social contract in which patients both contribute personal clinical data and benefit from the knowledge gained through the collaboration… Patient advocacy can best ensure that policy-makers in the U.S. Congress and elsewhere understand that well-intended efforts to guard patient privacy could impede the kind of data sharing required to accelerate the cures all are awaiting.

I see no alternative to a patient-centered approach. We must dismantle the paternalistic system administered by self-interested and self-appointed protectors of research subjects. Increasingly, what these parties actually protect patients from are the potential benefits of a system in which precompetitive information flows freely from bedside to lab bench (or computer terminal!) and then, largely via commercial enterprises, back to the bedside again. I am not advocating relieving institutions of the responsibility to guarantee that research subjects are fully informed and that the studies they enroll in are safely and expertly carried out. However, institutions should focus on protecting patients from procedural harm and careless handling of their medical records, not from hypothetical informational risks: if blood is to be drawn, the quantity should be reasonable and the draw conducted professionally; if images are to be acquired, associated risks should be clearly explained and the images competently acquired and interpreted; electronic medical records should be maintained in standard formats and stored on secure computer systems. However, if I, as an independent researcher with no ties to the initial study in which a patient enrolled, want access to raw data about the patient, my request should go directly to the patient, or to his or her designated agent, not to the researchers who carried out the original study or the institutions that employ them.

Fortunately, there is already significant experience designing systems that work this way [13, 15–17]. What we need is the will to use them and the vision to understand the central role they could play in coupling basic science to medicine. To make a patient-centered system work on the scale required, we would need to foster the development of a new type of organization, which Erlich et al. have dubbed the “trusted mediator” [17]. The trust in question is between the research participant and the mediator; the mediation is between the research participant and researchers who want access to the participant’s data. In many cases, existing patient-advocacy organizations could expand their missions to play this role. Over the long haul, a variety of trusted-mediation models would undoubtedly emerge since the needs of different classes of research participants would vary greatly; for example, patients suffering from a rare genetic disease that manifests itself at or soon after birth would require different protections than healthy adults who simply want to contribute their data to the general good. Competition between trusted-mediator organizations should be encouraged, and patients should be free to transfer their loyalties from one to another at any time. The key point is to eliminate the conflicts of interest that corrupt the current system. The fiduciary duty of an idealized trusted mediator should be to a single individual, the research participant whose data are at issue.

Of course, as databases grow to include information about millions of individuals, grouping of individuals into classes is inevitable. Most research participants would be willing to select one of a modest number of standard protocols, ranging from unrestricted sharing to case-by-case consideration of data access requests. For large studies, this system could evolve into a far more efficient system for assembling research subjects than current methods since subject acquisition would largely be a matter of computers talking to other computers. The sensitive steps involved in informing subjects of the risks and benefits of participation and allowing them time to consider their personal preferences would have already occurred before any particular study entered the picture.

Through social media, research participants could discuss their experiences with other individuals who are considering adopting particular sharing protocols. Given the scarcity of documented instances of informational harm to research participants, a reasonable expectation is that these discussions would often reassure newcomers that even the most permissive protocols pose minimal risk. Peer counseling would be particularly important in shaping the willingness of research participants to be recontacted by researchers for specific purposes. We have inflated recontact of research subjects into an unnecessarily vexing issue. It should become a routine option in all research studies. The key to making it one would be to channel recontact requests through trusted mediators. These mediators would know which option patients have chosen from a menu of choices. For example:

- “Allow researchers to do anything they want with the data they already have but leave me alone.”
- “Screen recontact requests and pass along the ones my physician thinks might be relevant to my health or that of my family.”
- “Subject recontact requests to peer review and approve the ones judged to have high scientific merit; and, by the way, do not recontact me more often than once a year!”

As population-genomic studies come to encompass millions of individuals, all of whom have had full genome sequences, recontact could become the standard method of recruiting subjects for specialized studies, including clinical trials. In populations of this size, it would become possible to acquire subjects by genotype, even for studies of rare genetic diseases. We know that the current system of acquiring subjects by phenotype is distorting our view of phenotype-genotype correlations, and it is time to act on this knowledge [18].

Importantly, the system I describe would be self-correcting since there is strength in numbers. If research participants become unhappy with the way their data are being managed, they would be able to demand and obtain changes in data-sharing protocols. As just one example, some groups of patients may want proposals for access to their data to be reviewed in particular ways. For instance, they might believe that review committees should have stronger participation by patients, ethicists, health economists, or other stakeholders and sources of expertise. There is every reason to think that trusted-mediator organizations would be more responsive to these requests than the institutional bureaucracies that now assess both the scientific merits and ethical acceptability of research studies. If different legal protections than those now in place prove necessary, legislators would hear from groups of patients who are well organized and in communication with one another through social media. None of this happens effectively now since, in a paternalistic model, protections are designed by researchers and institutions, not by the people who need and deserve protection.

Who should pay for this system? This vexing question is best approached by elimination. Obviously, we are not going to charge research participants for sharing their data. Public subsidies would be welcome, and perhaps essential, but it would be politically unrealistic and ethically problematic to socialize this system. Among other difficulties, over-reliance on public support would guarantee sequestration of data into geopolitically demarcated silos and bureaucratization of their administration. Privatization schemes in which data are provided to researchers for free should be viewed with suspicion: most such schemes are thinly veiled bait-and-switch inducements to use a company’s products. If we eliminate research participants, public agencies, and private companies as payers, we are left with user fees. Data about the genotypes, phenotypes, environmental exposures, and health of enormous numbers of individuals are becoming an essential research resource. Just as laboratories, libraries, and accounting systems are accepted costs of doing research, access to these data should be viewed similarly. Within a few decades, access to the information commons will overshadow the importance of all other research resources. The approach to managing these data proposed here would allow recapture of costs by patient-centered organizations, while leaving in place the sacrosanct principle that individuals should not be paid to participate in research studies except under exceptional circumstances. In contrast, the privatized alternative I sketched out above would almost certainly lead to bidding wars for enrollment of particularly valuable research subjects and routine payments to millions of others to insure their ongoing loyalty to particular data aggregators.

### Conclusion

Human genomics is at a crossroads. It may even be a bit past the crossroads, but I see little likelihood that any of the routes we are presently following will take us where we want to go. Fortunately, these are still early days in the mammoth undertaking of attempting to understand the interplay between human phenotypes, genotypes, and environmental exposures and to use what we learn to improve health. Humans are an outbred species with a complex population structure that is in the process of breaking down. We can, for the most part, only study ourselves observationally. Despite these obstacles, we are seeking to address a whole nexus of issues that have perplexed biologists since Darwin, Mendel, and the architects of the New Synthesis framed questions that have now moved to center stage in biomedical research. We should envision this undertaking as trans-generational. On this point, I quote from the NRC’s *Precision Medicine* report (full disclosure—I wrote this passage myself):

In a sense, this challenge has parallels with the building of Europe’s great cathedrals–studies started by one generation will be completed by another, and plans will change over time as new techniques are developed and knowledge evolves. As costs in the health care system are increasingly dominated by the health problems of a long-lived, aging population, one can imagine that [only] studies that last 5, 10, or even 50 years can answer many of the key questions on which clinicians will look to researchers for guidance. Many patients are already put on powerful drugs in their 40s, 50s, and 60s that they will take for the rest of their lives. The very success of some cancer treatments is shifting attention from short-term survival to the long-term sequelae of treatment. For all these reasons, the era during which a genetic researcher simply needed a blood sample and a reliable diagnosis is passing [19].

We must avoid cathedral-building initiatives that lead to bureaucratic science or premature commercialization. I have attempted to sketch an alternative future that preserves the ability of individual investigators and small laboratories to tap directly into a truly communal resource. Anyone who thinks we can do better by turning this project over to large, tightly managed teams analyzing their own siloed data sources, ignores the lessons of history. The policies I advocate will take time to put in place. At present, priority should go to medium-scale pilot projects, not to building large cohorts under hastily constructed rules of engagement. We need to hunker down for the long haul. The future of health care does lie with increasingly precise targeting of therapies to the right patients. Progress toward this future will require the best basic and applied science we can muster. The place to start in mobilizing this effort is by paying close attention to how we handle the data.

## Abbreviations

HGP: Human Genome Project
IT: Information technology
NIH: National Institutes of Health
